# Evaluation of the effects of fucoidan extracted from sargassum angustifolium on coagulation factors and biochemical parameters in male Wistar rats

**DOI:** 10.1016/j.htct.2025.106073

**Published:** 2025-10-30

**Authors:** Asma Dehghani, Ameneh Khoshvaghti, Narges Obeidi

**Affiliations:** a Department of Clinical sciences, Kaz. C., Islamic Azad University, Kazerun, Iran; b Department of Clinical Sciences, Faculty of Veterinary Medicine, Kazerun Branch, Islamic Azad University, Kazerun, Iran; c Department of Hematology, School of Paramedicine, Bushehr University of Medical Sciences, Bushehr, Iran

**Keywords:** Fucoidan, Sargassum angustifolium, Coagulation, Glucose metabolism, Iron homeostasis

## Abstract

**Introduction:**

This study investigates the effects of fucoidan extracted from Sargassum angustifolium on coagulation factors and biochemical parameters in male Wistar rats. Fucoidan, a sulfated polysaccharide from brown algae, is known for its anticoagulant, anti-cancer, and antioxidant properties

**Methods:**

The study involved 25 rats, divided into control, sham, and three experimental groups, receiving varying doses of fucoidan (100, 150, and 200 mg/kg body weight) over 28 days. The research focused on Prothrombin Time, Thrombin Time, and Partial Thromboplastin Time, along with biochemical markers like glucose, total protein, iron-related parameters, and albumin

**Results:**

This study found that fucoidan administration did not significantly affect the hemostasis tests, suggesting minimal impact on coagulation pathways in vivo. However, a dose-dependent reduction in glucose levels was observed, highlighting the potential of fucoidan as a hypoglycemic agent. Additionally, significant increases in transferrin, iron, and ferritin levels were noted, implying enhanced iron absorption and storage

**Conclusion:**

The findings underscore the therapeutic potential of fucoidan, particularly in managing glucose metabolism and iron homeostasis, while its minimal anticoagulant effect suggests safe usage in clinical settings where anticoagulation is undesirable. Further research is recommended to explore the full clinical benefits of fucoidan.

## Introduction

Seaweeds have long been a dietary staple in many East Asian cultures, revered not only for their culinary versatility but also for their rich nutritional profiles. These marine organisms are abundant in soluble dietary fibers, proteins, minerals, vitamins, antioxidants, phytochemicals, unsaturated fatty acids, and various bioactive compounds. Recent studies have further illuminated the potential health benefits of seaweeds, highlighting their roles in reducing inflammation, preventing blood clots, combating obesity, and lowering blood pressure [1,2]. Beyond these traditional applications, seaweeds are increasingly being explored for their therapeutic potential in addressing serious conditions such as cancer, allergies, diabetes, oxidative stress, and degenerative diseases [3, 4, 5].

Among the diverse types of seaweeds, brown algae (Phaeophyceae) are particularly noteworthy. This group includes approximately 2000 species across 265 genera, such as *Ascophyllum, Macrocystis, Laminaria, Eclonia, Sargassum*, and *Fucus* [6]. A key compound in brown algae is fucoidan, a sulfated polysaccharide found in their cell walls. Fucoidan has garnered significant attention due to its wide range of biological activities, including anticoagulant, antiviral, anticancer, antitumor, anti-inflammatory, and antioxidant effects [7].

The coagulation process, which can lead to conditions such as heart attacks and strokes, is a major health concern globally. The formation of blood clots involves a complex cascade of enzymatic events, beginning with the activation of proenzymes and culminating in the conversion of fibrinogen to fibrin by thrombin [8]. Heparin, a widely used anticoagulant, functions by inhibiting several key factors in this coagulation cascade via antithrombin III. Despite its effectiveness, heparin is associated with a range of side effects, including bleeding, heparin-induced thrombocytopenia (HIT), eosinophilia, skin reactions, and disturbances in liver function [9,10].

Fucoidans, complex sulfated polysaccharides primarily derived from brown seaweeds, have been studied for their anticoagulant properties. Initial investigations suggested potent anticoagulant activity similar to that of heparin. However, subsequent studies have revealed that the effects of fucoidan on coagulation may be more nuanced, influenced by factors such as molecular weight, sulfation pattern, and the source of extraction [11,12].

Among its most notable therapeutic effects are its anti-cancer and antioxidant activities. Fucoidan has demonstrated the ability to induce apoptosis, inhibit tumor growth, and prevent metastasis in various cancer models, making it a promising candidate for cancer therapy. Its antioxidant properties, on the other hand, are crucial in combating oxidative stress-related diseases by scavenging free radicals and enhancing the body's endogenous antioxidant defenses. The anticoagulant effect raises concerns about the safety of fucoidan, particularly when used in conditions where normal blood clotting is essential [13, 14, 15, 16].

Of fucoidan anticoagulant activity is primarily attributed to its structural characteristics, particularly the degree of sulfation and the presence of specific functional groups such as fucose, uronic acid, and sulfate. The anticoagulant effect of fucoidan is known to occur through several mechanisms, including the potentiation of antithrombin III activity, inhibition of thrombin generation, and interference with the binding of coagulation factors to cell surfaces. The sulfated groups in fucoidan molecules mimic the negative charge of heparin, allowing them to interact with antithrombin III and enhance its inhibitory effect on thrombin and factor Xa, crucial components in the blood coagulation cascade [17,18].

However, despite the extensive in vitro evidence of fucoidan anticoagulant activity, clinical studies have provided a different perspective. In human trials, the anticipated anticoagulant effects of fucoidan have not been consistently observed, and in many cases, fucoidan has been found to have minimal or no significant impact on the coagulation process. These findings suggest that the anticoagulant properties of fucoidan, while evident in controlled laboratory settings, may not translate to a clinical context, where complex physiological factors come into play [19].

This study aims to evaluate the effects of fucoidan extracted from *Sargassum angustifolium* on specific coagulation factors—Prothrombin Time (PT), Thrombin Time (TT), and Partial Thromboplastin Time (PTT)—as well as on biochemical parameters including total protein, glucose, transferrin, iron, ferritin, and albumin in male Wistar rats. By investigating these parameters, this research seeks to elucidate the potential of fucoidan as a therapeutic agent with a dual impact on coagulation and metabolic pathways. The results will provide valuable insights into the safe and effective use of fucoidan in clinical settings, particularly in managing complex metabolic conditions such as diabetes and anemia, while also addressing concerns related to its anticoagulant effects.

## Materials and methods

### Animal preparation and grouping

This study was conducted using 25 male Wistar rats, which were transferred to the animal care facility at the Islamic Azad University, Kazerun campus. Upon arrival, the rats were allowed to acclimatize to the laboratory environment for one week under standard housing conditions. These conditions included a controlled light-dark cycle (12:12 h), a temperature of 22 ± 2 °C, and consistent ventilation. During the acclimation period, the rats were provided with free access to a standard laboratory diet and water ad libitum. All rats received equal portions of the same standard diet at fixed times each day to minimize variations in glucose levels due to dietary factors.

Following acclimatization, the rats were randomly assigned to one of five groups, with each group housed individually in labeled cages to ensure accurate identification and monitoring throughout the study:

Control Group: Consisting of five rats, this group received only the regular diet without any additional treatment. Sham Group: Also consisting of five rats, this group received the regular diet plus 1 cc of distilled water administered orally (gavaging) daily for 28 days. This group served to control for any potential effects of the gavaging procedure. Experimental Group 1: This group included five rats that were administered 100 mg/kg body weight (BW) of fucoidan orally (gavaging) daily for 28 days along with the regular diet. Experimental Group 2: Consisting of five rats; this group received 150 mg/kg BW of fucoidan orally (gavaging) daily for 28 days in addition to the regular diet. Experimental Group 3: This group included five rats that were administered 200 mg/kg BW of fucoidan orally (gavaging) daily for 28 days along with the regular diet [20,21].

### Blood sample collection and coagulation factor analysis

Blood samples were collected from each rat on day 28 at the end of the study period. The blood was drawn via tail vein puncture under light anesthesia to minimize stress. Each blood sample was immediately placed on ice and transported to the laboratory for coagulation factor analysis.

The coagulation parameters analyzed included Prothrombin Time (PT), Partial Thromboplastin Time (PTT), and Thrombin Time (TT). These parameters were measured using standard coagulation assays to assess the impact of fucoidan on the extrinsic, intrinsic, and common pathways of the coagulation cascade.

### Fucoidan extraction

The fucoidan was extracted from the brown algae *Sargassum angustifolium*, which was collected from the shores of Bushehr, Iran. The algae were identified at the Persian Gulf Research Institute, washed with seawater, and cleaned of debris and other impurities. The samples were dried in the shade for four days and stored in zip-lock plastic bags.

For extraction, 20 *g* of algae were mixed with 400 mL of distilled water and heated at 45 °C for 45 min on a shaker. Sodium chloride (NaCl, 1 g) was added to adjust the pH to 7. The pH was then adjusted to 7.5 with 0.1 M NaOH, and the mixture was kept at 45 °C for 3 h. After an hour, 24 *g* of NaCl were added. The resulting compounds were precipitated by adding 100 mL of absolute ethanol and left at room temperature overnight. The compounds were washed twice daily for two days with 500 mL of distilled water for 30–60 min on a shaker at room temperature.

On the second day, the mixture was centrifuged, and the supernatant was removed. An additional 100 mL of absolute ethanol was added and kept overnight at room temperature. On the third day, the mixture was filtered, and the remaining filtrate was dissolved in 150 mL of distilled water and incubated for an hour at 40 °C. The pH was adjusted to 3 with HCl and filtered using a 0.2-micron filter. The solution was then lyophilized to obtain fucoidan powder.

### Biochemical analysis

Biochemical analysis of fucoidan, including monosaccharide composition and structural characteristics, was performed using high-performance liquid chromatography (HPLC) and Fourier-transform infrared spectroscopy (FTIR). The hydrolyzed polysaccharide sample (90 min in 2 M trifluoroacetic acid at 120 °C) was injected into the HPLC system (VARIAN, Pro Star, USA) using a mobile phase of acetonitrile/deionized water (90:10) at a flow rate of 2 mL/min. FTIR analysis (PerkinElmer FT-IR, Spectrum RXI, USA) was performed on the ground sample in potassium bromide (KBr), with signals collected automatically using 60 scans in the range of 4000–400/cm with a resolution of 32/cm.

### Preparation of fucoidan solutions

One gram of fucoidan powder was dissolved in 50 mL of distilled water and placed on a shaker at 25 °C for 12 h. The resulting solution (0.02 g/mL) was filtered through a 0.4-micron filter and used for coagulation assays.

### Coagulation assays

Prothrombin Time (PT): 200 µL of PT solution (thermo scientific) was warmed to 37 °C in a water bath. An aliquot of 100 µL of fresh plasma was added to test tubes containing 200 µL of PT solution, 190 µL of PT solution plus 10 µL of normal saline, and 190 µL of PT solution plus 10 µL of fucoidan. The coagulation time was measured as the time taken for fibrin formation.

Partial Thromboplastin Time (PTT): 100 µL of PTT solution (thermo scientific) was warmed to 37 °C in a water bath. An aliquot of 100 µL of fresh plasma was added to test tubes containing 90 µL of PTT solution, 90 µL of PTT solution plus 10 µL of normal saline, and 90 µL of PTT solution plus 10 µL of fucoidan. After 2 min, 100 µL of 37 °C calcium chloride solution was added and the coagulation time was measured.

Thrombin Time (TT): Plasma was mixed with bovine thrombin reagent containing bovine albumin, calcium chloride, and buffer. The clotting time was measured optically at a wavelength of 405 nm.

### Biochemical analysis of blood samples

Total Protein: Total protein levels were measured using a standard Biuret method. Plasma samples were mixed with Biuret reagent and incubated at 37 °C for 10 min. The optical absorbance was then measured at 540 nm using a microplate reader (BioTek, USA). The total protein concentration was calculated based on a standard curve prepared using bovine serum albumin (BSA).

Glucose: Glucose levels were determined using a glucose oxidase-peroxidase (GOD-POD) method. Plasma samples were incubated with glucose oxidase and peroxidase enzymes at 37 °C for 15 min, and the colorimetric reaction was measured at 505 nm. A glucose standard solution was used to generate a standard curve for glucose quantification.

### Iron-Related parameters

Transferrin: Serum transferrin levels were measured using an enzyme-linked immunosorbent assay (ELISA) kit (Azma plast), following the manufacturer’s instructions. Optical absorbance was read at 450 nm, and transferrin concentration was calculated based on the standard provided with the kit.

Iron (Fe): Serum iron concentration was measured using a colorimetric iron assay kit (Delta.dp). The plasma samples were mixed with a chromogen that binds iron to form a colored complex, and optical absorbance was measured at 562 nm.

Ferritin: Ferritin levels were determined using an ELISA kit (Delta.DP), following the manufacturer's protocol. Optical absorbance was read at 450 nm, and ferritin concentration was calculated from a standard curve.

Albumin: Plasma albumin levels were measured using the bromocresol green (BCG) method. Plasma samples were mixed with BCG reagent, and optical absorbance was measured at 630 nm using a microplate reader. Albumin concentration was determined using a standard curve prepared with known albumin standards.

Transferrin Saturation: Transferrin saturation percentage was calculated using the following formula:

$$Transferrin\;Saturation\;\left(\%\right)=\left(\frac{serum\;iron}{total\;iron\;binding\;capacity\;\left(TIBC\right)}\right)*100$$

TIBC=Transferrin×1.25

Serum iron and TIBC levels were measured as described previously, and transferrin saturation was determined accordingly.

## Results

The effects of fucoidan extracted from Sargassum angustifolium on coagulation factors and biochemical parameters were evaluated in male Wistar rats.

### Coagulation factors

#### Prothrombin time (PT)

**Figure 1:**
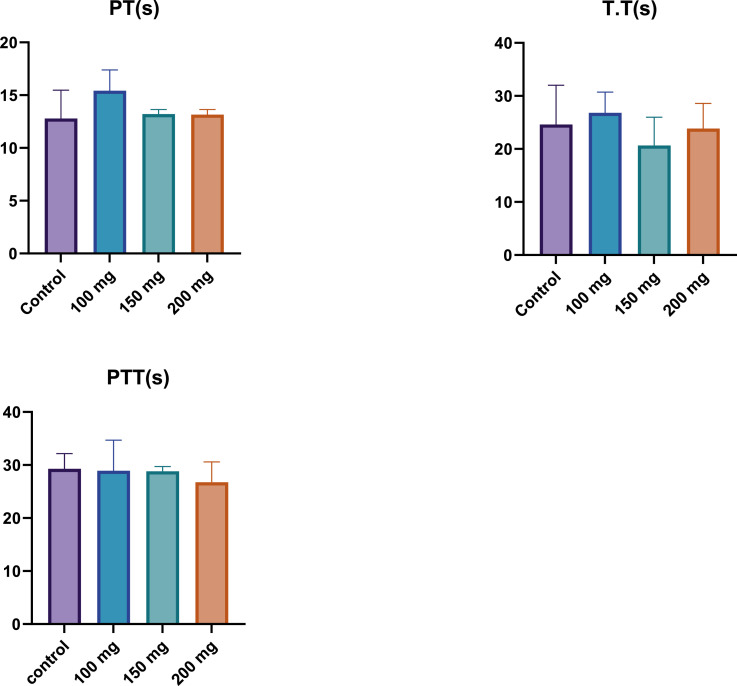
Prothrombin Time (PT), Thrombin Time (TT), and Partial Thromboplastin Time (PTT) in male Wistar rats after 28 days of fucoidan administration. The results show no statistically significant changes in PT, TT, or PTT across different fucoidan dosages (100, 150, and 200 mg/kg BW), indicating that fucoidan does not significantly alter the coagulation pathways.

The PT is a critical parameter used to evaluate the extrinsic pathway of coagulation. In this study, PT values were measured across the control group and the fucoidan-treated groups, which received varying doses of fucoidan. As illustrated in Figure 1, while there were slight variations in PT across the different groups, these differences were not statistically significant. The 200 mg/kg BW fucoidan group exhibited a marginally higher PT compared to both the control group and the lower-dose fucoidan-treated groups. However, the overall consistency in PT values suggests that fucoidan, at the doses administered, does not significantly alter the extrinsic coagulation pathway.

#### Thrombin time (TT)

TT is a measure of the time it takes for thrombin to convert fibrinogen into fibrin, an essential step in the final stages of the coagulation cascade. The TT results, as shown in Figure 1, indicated minimal variations between the control group and the fucoidan-treated groups. All groups exhibited similar TT values, indicating that fucoidan administration did not substantially influence this coagulation parameter. This finding suggests that fucoidan does not significantly affect the thrombin-mediated conversion of fibrinogen to fibrin, an essential component of blood clot formation.

#### Partial thromboplastin time (PTT)

The PTT assesses the intrinsic and common pathways of coagulation. As displayed in Figure 1, the control group exhibited the highest PTT value, while the fucoidan-treated groups showed a slight, dose-dependent decrease in PTT. Among the fucoidan-treated groups, the 200 mg/kg group demonstrated the lowest PTT value, indicating a modest reduction in the time required for clot formation via the intrinsic pathway. Despite these observations, the differences between the groups were modest and may not be statistically significant. Therefore, while fucoidan administration appears to exert a mild influence on the intrinsic coagulation pathway, this effect is not pronounced.

### Biochemical parameters

#### Total protein

**Figure 2:**
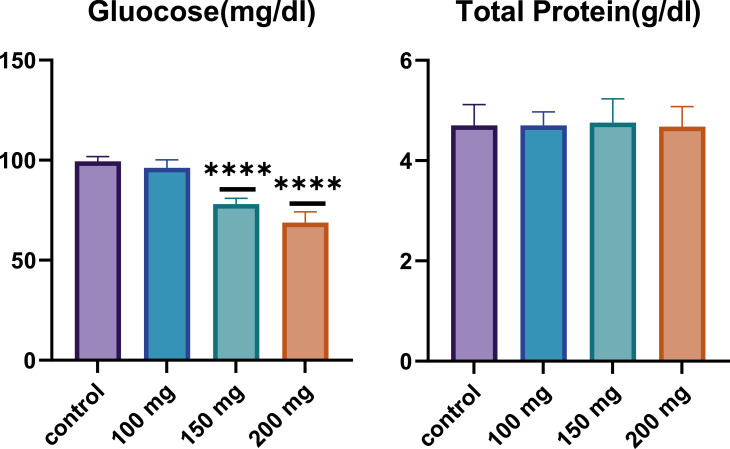
Effect of Fucoidan on glucose and total protein levels in male Wistar rats after 28 days of fucoidan administration. A significant dose-dependent decrease in glucose levels is observed, particularly at 150 mg/kg and 200 mg/kg BW (p-value < 0.0001). Total protein levels remain consistent across all groups, indicating no significant impact on overall protein metabolism. ****: p-value < 0.0001.

Total protein levels are an essential indicator of overall health and nutritional status, reflecting the balance between protein synthesis and degradation. As shown in Figure 2, total protein levels were measured across the control group and the fucoidan-treated groups. The results indicated comparable protein levels for all groups, with no substantial differences observed between the control group and the groups treated with various fucoidan dosages. This finding suggests that fucoidan administration does not significantly impact overall protein metabolism or synthesis in the rats, maintaining a consistent total protein concentration across different treatment groups.

#### Glucose

**Figure 3:**
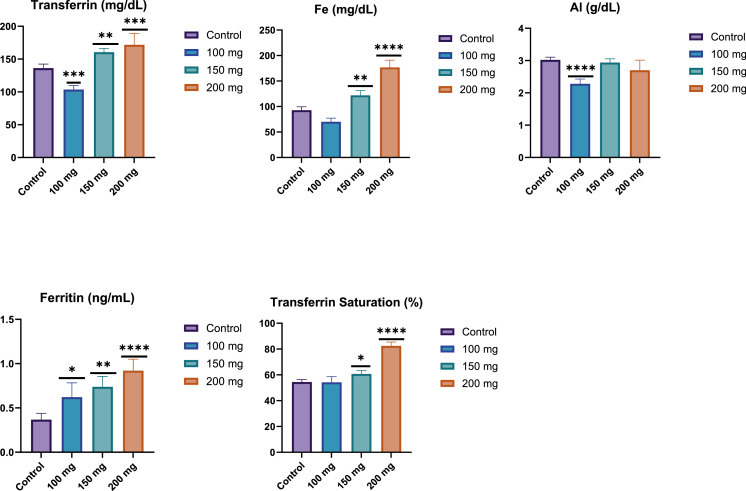
Effect of fucoidan on iron-related parameters (Transferrin, iron (Fe), ferritin, Transferrin Saturation and albumin levels) in male Wistar rats after 28 days of fucoidan administration. Fucoidan treatment resulted in a dose-dependent increase in transferrin, iron, and ferritin levels, suggesting enhanced iron absorption and storage. Albumin levels decreased at the 100 mg/kg dose, with no significant changes at higher doses, indicating a complex effect of fucoidan on protein metabolism. ****: p-value <0.0001; ***: p-value <0.001; **: p-value <0.01; *: p-value <0.05.

Glucose levels were also assessed as a key biochemical parameter, given the known metabolic effects of fucoidan. As presented in Figure 3, a notable dose-dependent decrease in glucose levels was observed in the fucoidan-treated groups compared to the control group. Specifically, the 150 mg/kg and 200 mg/kg BW fucoidan-treated groups exhibited statistically significant reductions in glucose levels (p-value < 0.0001) compared to the control group. The 200 mg/kg BW group showed the most pronounced decrease in glucose concentration, highlighting the potential of fucoidan as a hypoglycemic agent. This dose-dependent hypoglycemic effect suggests that fucoidan may influence the glucose metabolism, potentially offering therapeutic benefits for conditions characterized by elevated blood glucose levels, such as diabetes (Figure 2).

#### Iron-Related parameters

Transferrin: The graph shows a dose-dependent increase in transferrin levels with increasing fucoidan dosage. The 200 mg/kg BW group exhibited significantly higher transferrin levels (p-value <0.001) compared to the control group. The 100 mg/kg BW group showed a slight decrease, while the 150 mg/kg and 200 mg/kg BW groups demonstrated progressive increases in transferrin concentration.

Iron (Fe): The results for serum iron (Fe) levels showed a significant increase in response to fucoidan administration in a dose-dependent manner. Specifically, the group receiving 150 mg/kg BW of fucoidan exhibited a statistically significant increase in serum iron levels compared to the control group (p-value <0.01). Furthermore, the 200 mg/kg BW dose resulted in an even more pronounced elevation in serum iron levels, with a highly significant difference compared to the control group (p-value <0.0001). These findings suggest that fucoidan may enhance iron absorption and storage at higher doses (Figure 3).

Ferritin: Ferritin levels increased dose-dependently with fucoidan treatment. The 200 mg/kg BW group showed the most significant increase (p-value <0.0001), followed by the 150 mg/kg BW group (p-value <0.01), and the 100 mg group (p-value <0.05). This suggests that higher doses of fucoidan lead to greater ferritin production or storage (Figure 3).

Transferrin Saturation: A dose-dependent increase in transferrin saturation was observed across the experimental groups. The 150 mg/kg BW group showed a significant increase compared to the control group (p-value <0.05), while the 200 mg/kg group exhibited a highly significant rise (p-value <0.0001). No significant changes were noted in the 100 mg/kg BW group compared to the control. These findings suggest that higher doses of fucoidan may enhance iron transport and absorption (Figure 3).

Albumin: The graph shows changes in albumin levels across different fucoidan dosages. The 100 mg/kg BW fucoidan group demonstrated a significant decrease in albumin levels (p-value <0.0001) compared to the control. While the 150 mg/kg BW and 200 mg/kg BW groups also showed lower albumin values than the control, the differences were less pronounced and not marked as statistically significant in the graph. This suggests that fucoidan, particularly at lower doses, may influence albumin production or metabolism (Figure 3).

These results indicate that fucoidan administration has significant effects on iron metabolism and related parameters. The observed increases in transferrin, iron, and ferritin levels suggest that fucoidan may enhance iron absorption, transport, or storage in a dose-dependent manner.

## Discussion

A salient finding of this study is the significant, dose-dependent reduction in blood glucose levels following fucoidan administration. This observation aligns with emerging literature underscoring the antidiabetic potential of fucoidan. For instance, Kim et al. reported that fucoidan improved insulin sensitivity and reduced hyperglycemia in diabetic mouse models [22]. The underlying mechanisms are multifactorial, involving the modulation of key enzymes in glucose metabolism, enhancement of insulin signaling pathways, and improvement of pancreatic β-cell function [23].

The results of this study also revealed significant changes in iron-related parameters following fucoidan administration. A dose-dependent increase was observed in transferrin levels, with the 200 mg/kg BW group showing significantly higher concentrations compared to the control. Similarly, all fucoidan-treated groups exhibited elevated iron levels, with the most pronounced increases in the 100 mg/kg BW and 200 mg/kg BW groups. Ferritin levels also increased dose-dependently, with all treated groups showing significant elevations compared to the control.

The concurrent increase in transferrin and iron levels indicates enhanced iron transport capacity, which could be beneficial in conditions such as iron deficiency anemia. The increase in transferrin and decrease in albumin are particularly interesting, as both are considered ‘negative acute phase’ proteins; typically, these proteins decrease during inflammation. The lack of similar significant changes in albumin and transferrin levels in rats that received fucoidan probably reflects changes in iron homeostasis induced by fucoidan, not due of inflammation.

The liver plays a central role in iron metabolism, and the results of this study indicate that fucoidan may influence hepatic function related to iron handling. Increased iron levels stimulate the production of transferrin, which is primarily synthesized in the liver. This increase in transferrin production may be a compensatory mechanism to manage the elevated iron levels observed. The liver also regulates ferritin production, and the increased ferritin levels observed in this study further support the notion of enhanced iron storage capacity in response to fucoidan treatment [24].

Additionally, the observed changes in iron-related parameters suggest that fucoidan may enhance iron absorption in the intestine. The interaction of fucoidan with the intestinal epithelium could potentially modify the expression or activity of iron transporters, such as divalent metal transporter 1 (DMT1) or ferroportin, leading to increased iron uptake. This enhanced absorption could explain the elevated plasma iron levels observed across all fucoidan-treated groups. The dose-dependent increase in ferritin further supports this hypothesis, as increased iron absorption would necessitate greater iron storage capacity [25,26].

The findings of this study suggest that fucoidan supplementation significantly influences iron metabolism in a dose-dependent manner. The marked increases in transferrin, serum iron, and ferritin levels, particularly in the 150 mg/kg and 200 mg/kg BW groups, indicate that fucoidan enhances iron absorption, transport, and storage. The significant rise in transferrin saturation further supports this notion, suggesting improved iron bioavailability. Interestingly, while the 100 mg/kg group did not exhibit substantial changes in transferrin saturation or serum iron levels, the 150 mg/kg and 200 mg/kg groups showed progressive increases, highlighting the importance of dosage of fucoidan on iron homeostasis. These results align with previous studies that have suggested the potential role of polysaccharides in enhancing iron metabolism, further reinforcing the therapeutic potential of fucoidan in addressing iron deficiency.

The lack of a significant increase in albumin, despite the elevation in other liver-produced proteins like transferrin, warrants further investigation. This discrepancy might be due to the differential effects of fucoidan on various liver functions or could indicate a complex interplay between iron metabolism and overall protein synthesis in the liver.

The anticoagulant properties of fucoidan have been widely studied, particularly in vitro, where it has shown significant potential to inhibit various steps of the coagulation cascade. These effects are largely attributed to the structural features of fucoidan, such as its sulfation pattern and molecular weight, which allow it to interact with key proteins like thrombin and antithrombin III, thereby modulating blood coagulation [27,28]. However, the results from this study, along with emerging in vivo data, suggest that the anticoagulant efficacy of fucoidan observed in vitro does not necessarily translate to a significant impact in vivo. However, it is important to note that not all species of brown algae possess anticoagulant properties. Some species, as mentioned in related literature, lack these effects, suggesting that the anticoagulant potential may vary significantly depending on the species and specific composition of fucoidan [29,30].

The findings of the present study demonstrated that fucoidan, at the administered doses, did not significantly alter prothrombin time (PT), thrombin time (TT), or partial thromboplastin time (PTT) in Wistar rats. This discrepancy between in vitro and in vivo effects might be due to several factors. In the complex biological environment of a living organism, fucoidan may undergo metabolic modifications that reduce its interaction with coagulation factors. Additionally, the bioavailability of fucoidan and its distribution within the body could limit its ability to reach effective concentrations at sites where coagulation occurs.

It is worth noting that our study utilized lower doses of fucoidan (100, 150, and 200 mg/kg BW) compared to some previous studies that have used very high doses (1000 and 1500 mg/kg BW). Our decision to reduce the dose was based on the principle of finding the minimal effective dose that could produce beneficial effects while minimizing potential side effects. The significant changes observed in glucose levels and iron-related parameters at these lower doses suggest that fucoidan can exert physiological effects even at more moderate concentrations.

The absence of a pronounced anticoagulant effect in vivo paves the way for the use of fucoidan in therapeutic contexts where anticoagulation is undesirable. For instance, the lack of significant interference of fucoidan with blood clotting makes it a promising candidate for cancer treatment, where anticoagulation might pose risks. Fucoidan has been recognized for its potential anti-cancer properties, including its ability to induce apoptosis, inhibit tumor growth, and reduce metastasis through various mechanisms. The minimal impact on coagulation parameters ensures that fucoidan could be safely incorporated into treatment regimens without exacerbating bleeding risks.

Furthermore, the anti-inflammatory and antioxidant properties of fucoidan add another layer of therapeutic potential, especially in the context of chronic diseases such as cancer. Fucoidan has been shown to modulate inflammatory pathways, reducing the production of pro-inflammatory cytokines and inhibiting the activation of key inflammatory cells. This, combined with its ability to scavenge free radicals and protect cells from oxidative stress, highlights the role of fucoidan as a multi-functional therapeutic agent [29].

In summary, while fucoidan from *Sargassum angustifolium* at doses of 100, 150, and 200 mg/kg BW may not exhibit significant in vivo anticoagulant effects, its safety profile regarding coagulation, along with its beneficial properties, including hypoglycemic, anti-cancer, anti-inflammatory, and antioxidant activities, positions it as a valuable compound for the development of novel therapeutic strategies. Further research, particularly clinical studies, is warranted to explore the full spectrum of the benefits of fucoidan in human health.

## Data availability statement

The data supporting the findings of this study, including raw datasets, figures, and supplementary materials, are available from the corresponding author, Ameneh Khoshvaghti, upon reasonable request. Due to confidentiality agreements, some data may not be publicly accessible, but inquiries regarding specific data points or methods can be directed to the corresponding author.

## Conflicts of interest

The authors declare no conflicts of interest.
